# Multimodal evoked potentials for functional quantification and prognosis in multiple sclerosis

**DOI:** 10.1186/s12883-016-0608-1

**Published:** 2016-06-01

**Authors:** Xavier Giffroy, Nathalie Maes, Adelin Albert, Pierre Maquet, Jean-Michel Crielaard, Dominique Dive

**Affiliations:** Department of Neurology, University Hospital of Liege, Rue Grandfosse 31-33, 4130 Esneux, Belgium; Department of Physical Medicine and Rehabilitation, University Hospital of Liege, B35, 4000 Liege, Belgium; Department of Biostatistics and Medico-Economic Information, University Hospital (CHU, ULg) of Liege, B35, 4000 Liège, Belgium

**Keywords:** Evoked potentials, Multiple sclerosis, Prognosis, Disability

## Abstract

**Background:**

Functional biomarkers able to identify multiple sclerosis (MS) patients at high risk of fast disability progression are lacking. The aim of this study was to evaluate the ability of multimodal (upper and lower limbs motor, visual, lower limbs somatosensory) evoked potentials (EP) to monitor disease course and identify patients exposed to unfavourable evolution.

**Methods:**

One hundred MS patients were assessed with visual, somatosensory and motor EP and rated on the Expanded Disability Status Scale (EDSS) at baseline (T0) and about 6 years later (T1). The Spearman correlation (r_S_) was used to evaluate the relationship between conventional EP scores and clinical findings. Multiple (logistic) regression analysis estimated the predictive value of baseline electrophysiological data for three clinical outcomes: EDSS, annual EDSS progression, and the risk of EDSS worsening.

**Results:**

In contrast to longitudinal correlations, cross-sectional correlations between the different EP scores and EDSS were all significant (0.33 ≤ r_S_ < 0.67, *p* < 0.001). Baseline global EP score and EDSS were highly significant predictors (*p* < 0.0001) of EDSS progression 6 years later. The baseline global EP score was found to be an independent predictor of the EDSS annual progression rate (*p* < 0.001), and of the risk of disability progression over time (*p* < 0.005). Based on a ROC curve determination, we defined a Global EP Score cut off point (17/30) to identify patients at high risk of disability progression illustrated by a positive predictive value of 70 %.

**Conclusion:**

This study provides a proof of the concept that electrophysiology could be added to MRI and used as another complementary prognostic tool in MS patients.

**Electronic supplementary material:**

The online version of this article (doi:10.1186/s12883-016-0608-1) contains supplementary material, which is available to authorized users.

## Background

Evoked potentials (EP) provide quantitative functional measures in well-defined pathways of the central nervous system (CNS). In multiple sclerosis (MS), the use of EP to diagnose the disease or assess its biological activity has been limited, mainly because of the high sensitivity of magnetic resonance imaging (MRI), especially at the brain level [1]. There are situations though where electrophysiological data can provide useful diagnostic information. For instance, visual EP can demonstrate lesional spatial dissemination [2, 3]. During the course of MS, EP can be used to confirm unclear relapses in patients expressing vague or transitory symptoms [4]. EP have also been used in some clinical trials targeted on relapse anti-inflammatory treatments [5], immunomodulatory disease modifying drugs [6–8] and symptomatic medications designed to facilitate the central nervous conductions [9, 10].

Overall, EP were more sensitive than clinical metrics to demonstrate drug efficacy [5, 6], anticipate clinical changes [6], and help predicting good response in specific situations [9, 10].

If conventional MRI (T1, T2 and T1 + Gadolinium) and clinical evaluation can be regarded as the standard approach for diagnosing MS and monitoring its biological activity, they usually demonstrate a weak association known as the functional-anatomical paradox [11–13]. By contrast, EP can evaluate long eloquent pathways well correlated to the functional involvement [14]. EP latency and amplitude abnormalities evaluate the pathological conduction characteristics. Latency prolongation is due to demyelination, while conduction block or axonal loss lead to reduced amplitude [15]. In previous studies, cross-sectional correlations with EDSS were generally good, ranging from 0.3 to 0.6 [11, 16–22]. By contrast, longitudinal correlations were weaker [11, 17, 20, 23], which may be attributed to lower EP reproducibility [4, 24], to a level-off effect of EP measures in patients with more advanced disease [14], but also to a lack of responsiveness of clinical scales [25, 26]. The weak longitudinal association is balanced by the potential ability of EP to anticipate a clinical deterioration, which could be useful for the prognosis of the disease course [7, 16, 17, 27], especially in the era of early treatment procedure for patients with a poor prognosis. Yet, the prognostic role of EP is not fully admitted and further studies are needed to address this issue [28]. Therefore, the present work aimed to evaluate the ability of multimodal EP, summarized by a conventional score, to monitor MS and identify patients exposed to an unfavourable progression.

## Methods

### Subjects

We performed a retrospective analysis of the local database of 100 MS patients followed in our academic clinical centre between 2001 and 2014. The selection was based on a convenience sampling procedure. The inclusion criteria were: (1) a definite diagnosis of MS based on the 2001 and 2005 McDonald criteria [29, 30]; (2) a clinical follow-up of at least 3 years, including a disability rating using the Expanded Disability Status Scale (EDSS) obtained at baseline (T0) and at the end of the follow-up period (T1) [31]; (3) a multimodal EP examination at T0, i.e. motor evoked potentials from lower and upper limbs (MEP), visual evoked potentials (VEP), and somatosensory evoked potentials from lower limbs (SEP); (4) a clinically stable phase at the time of each evaluation ensured by a 3-month period between the previous relapse and the evaluation.

For 76 of the 100 MS patients, a second electrophysiological examination was available at the end of the follow-up period (T1) enabling a longitudinal analysis of multimodal EP data. The study was approved by the Local Ethics Committee (University Hospital of Liege) and has been performed in accordance with the ICH (International Council for Harmonisation of Technical Requirements for Pharmaceuticals for Human Use) guidelines.

### Evoked potentials

All electrophysiological examinations were performed by the same investigator (XG) using the same recording system (Keypoint, Alpine Biomed, France); clinical evaluations were conducted by a non-blinded investigator (DD).

Motor Evoked Potentials (MEP) were recorded bilaterally from the first dorsal interosseous (FDI) in the upper limbs and from the tibialis anterior (TA) in the lower limbs. Each measurement started by recording the compound muscle action potential (CMAP) elicited by a supramaximal electrical stimulation of the ulnar nerve at the wrist and common peroneal nerve at the knee. Then, three transcranial magnetic stimulations (TMS) were delivered to the hand and leg areas of the motor cortex, respectively on Cz and Fz, with a Magstim 200 device (The Magstim Company Ltd., Whitland, UK) using a circular coil (maximal output 2.2 T). For each limb, the shortest latency and highest amplitude event out of the 3 waves were selected for analysis. A peripheral magnetic stimulation was applied over the C8 and L5 nerve root for the upper limbs and lower limbs, respectively, to derive the central motor conduction time (CMCT). The CMCT was obtained by subtracting the peripheral conduction time obtained by magnetic stimulation of the cervical and lumbar spinal roots from the cortical MEP latency. The following MEP parameters were considered in the study: peripheral, radicular and cortical latencies; CMCT; absolute baseline-to-peak amplitude from the peripheral and cortical stimulations; amplitude ratio (AR) expressed as the MEP_ampl_/CMAP_ampl_ ratio; duration of the cortical stimulation induced response measured from the onset of the initial negative deflection to the latest positive deflection.

Visual evoked potentials (VEP) were recorded using EEG cup electrode Ag-AgCl electrodes. The active electrode was placed at Oz and the reference at Fz, based on the 10–20 international EEG system. Monocular visual stimulation was performed with a pattern-reversal check board screen. The size of the squares was adjusted to subtend a 15-min visual arc with an individual square. The two parameters used for the analysis were latency of the P100 component and N75-P100 amplitude difference.

Somatosensory evoked potentials (SEP) of the lower extremities were obtained through posterior tibial nerve electrical stimulation at the ankle. The afferent volley was recorded at 3 levels by active electrodes located at the popliteal fossa, over the intervertebral space D12-L1 and 2 cm posterior to Cz. The reference electrodes were placed, respectively, on the lateral side of the thigh, over the intervertebral space L5-S1 and at Fz. The parameters studied were the latencies of the main peripheral (N9), spinal (N21) and cortical (P40) components along with P40-N21 difference (central sensitive conduction time) and P40 amplitude (baseline-to-peak).

### Analysis and rating of EP data

We developed from EP data a conventional ordinal score (EP score) modified from Jung et al. [16]. Specifically, for the sensory EP, both latency and amplitude abnormalities were included in the score. For MEP, the duration of the cortex stimulation induced response was added to these parameters to account for the temporal dispersion of the corticospinal discharges. Absolute or body side difference values above 2 standard deviations (SD) from our local normative data were considered pathological.

For each modality and body side, the score was derived from a 6-point graded ordinal scale summarized in Table 1. A result in the normal range but with a pathological body side difference received a score of 1 (for latency) and 2 (for amplitude). This score was assigned to the worst body side. In case of absolute latency above the normal limit, we attributed a score of 2, 3 or 4, according to the delay. The worst and maximum score of the scale (5) was attributed to an evoked response either absent or with amplitude below the normal range. Hence, the EP score for each side and each modality (UL MEP, LL MEP, LL SEP, VEP) ranged from 0 to 5. To give equal weight to each modality in the global EP score (MEP + SEP + VEP), we divided the MEP score by 2 since it was derived from the 4 limbs (UL MEP + LL MEP). Thus, the overall worst score of the scale was 30.

**Table 1:** Description of the 6-point graded EP ordinal scale

EP results (MEP, SEP, VEP)	Score
Normal (ampl and lat)	0
Pathological body side difference lat	1
Pathological body side difference ampl or pathological lat but < P33.3	2
P33.3 < lat < P66.7 or increased duration of the cortical stimulation induced response (MEP)	3
P66.7 < lat	4
Pathological ampl and normal duration of the cortical stimulation induced response (MEP)	5

*EP* evoked potentials, *MEP* motor evoked potentials, *SEP* somatosensory evoked potentials, *VEP* visual evoked potentials, *Ampl* amplitude, *Lat* latency, P33.3, first tertile, derived from the MS sample presenting a pathological latency; P66.7, second tertile, derived from the MS sample presenting a pathological latency; MEP, motor evoked potentials

### Statistical analysis

Results were expressed as mean and SD for quantitative data and as frequencies (number and percent) for categorical findings. The associations between EP scores and clinical data were assessed by the Spearman correlation coefficient (r_s_). Mean values between groups (e.g. relapsing–remitting vs. progressive patients) were compared by analysis of variance (ANOVA) and proportions by the chi-square or Fisher exact test. Score changes between T0 and T1 were assessed by the paired Student t-test.

Multivariate methods were used to assess the 6-year predictive value of demographic (age, size, weight, sex, laterality), clinical (relapsing-remitting or progressive phenotype, disease duration, follow-up duration, disease-modifying therapy between T0 and T1) and baseline electrophysiological (global EP score) data for three main clinical outcomes: the EDSS score (model 1), the annual EDSS progression score (model 2), and the EDSS worsening (yes/no). EDSS worsening was defined as a 1-point increase from T0 to T1 given a baseline EDSS <5.5 or an increase of 0.5 point given a baseline EDSS ≥ 5.5 [17] (model 3). The EDSS progression score per year was obtained by the absolute EDSS progression score divided by the follow-up duration in years. Multiple regression was used for models 1 and 2 and logistic regression for model 3. The quality of the fit was measured by the coefficient of determination (R^2^) for models 1 and 2 and by the area under the curve (AUC) for model 3. Results were expressed in terms of regression coefficients with their standard error (SE). Odds Ratios (OR) and associated 95 % confidence intervals (95 % CI) were added for the logistic model. Observed and predicted probabilities of worsening were cross-classified to further enhance the prognostic ability of baseline EP data. Finally, the receiver operating characteristic (ROC) curve method was used to determine an optimal cut-off value on the total EP scale to discern patients with poor and favourable prognosis. Results were considered significant at the 5 % critical level (*p* < 0.05). All statistical calculations and graphs were performed with SAS (version 9.4 for Windows) and the R software (version 3.0.3).

## Results

Clinical and electrophysiological data are summarized in Table 2. At baseline, 90 patients were in a relapsing-remitting (RR) course, 9 patients in a secondary progressive (SP) course and one patient in a primary progressive (PP) course. The mean age was 39 years (SD 10) and the disease duration 9.1 years (SD 8.0). EDSS averaged 3.0 (SD 1.2) and the mean global EP score was 12.1 (SD 7.6). When comparing RR and P phenotypes, significant differences were observed for all clinical and electrophysiological parameters (except VEP) even after adjustment for disease duration.

**Table 2:** Mean (SD) of clinical and electrophysiological characteristics recorded at baseline

	All patients	RR	P	RR versus P	
	(*n* = 100)	(*n* = 90)	(*n* = 10)	p*	p**
F/M ratio	62/38	59/31	3/7	0.040	0.072
Age (yrs)	39 (10)	37 (9)	53 (7)	<0.0001	<0.0001
Disease duration (yrs)	9 (8)	8 (7)	19 (11)	0.0030	NA
EDSS (0.0–10.0)	3.0 (1.2)	2.8 (1.0)	5.0 (0.83)	<0.0001	<0.0001
Global EP score (/30)	12.1 (7.6)	11.2 (7.4)	19.8 (5.4)	0.0006	0.011
UL MEP score (/10)	2.6 (3.0)	2.3 (2.9)	5.0 (3.3)	0.0064	0.035
LL MEP score (/10)	3.9 (3.4)	3.5 (3.3)	7.4 (2)	0.0004	0.0057
LL SEP score (/10)	4.7 (3.4)	4.4 (3.4)	7.5 (1.8)	0.0047	0.020
VEP score (/10)	4.2 (3.6)	4.0 (3.5)	6.1 (3.9)	0.079	0.35

*MS* multiple sclerosis, *RR* relapsing-remitting, *P* progressive, *SD* standard deviation, *F* female, *M* male, *EDSS* expanded disability status scale, *EP* evoked potentials, *UL* upper limb, *LL* lower limb, *MEP* motor evoked potentials, *SEP* somatosensory evoked potentials, *VEP* visual evoked potentials, *NA* not applicable

*p-value of the comparison between relapsing and progressive courses; **p-value of the comparison between relapsing and progressive courses adjusted for disease duration

After a median follow-up period of 6.3 years, the deteriorations of EDSS (+0.6; SD 1.1) and of the global EP score (+2.7; SD 3.9) were highly significant (*p* < 0.0001), as were those of lower limbs MEP and SEP. By contrast, the visual functional system progression, although significant (*p* < 0.05), was less marked, while the sensory functional system did not evidence any change. In classifying clinical and EP data with respect to their relative progression over time, we found that the global and LL MEP scores presented a worsening nearly twice as high as clinical measures (Table 3).

**Table 3:** Clinical and EP data ranked according to their relative progression (*N* = 76 MS patients)

Score	T0	T1	T1–T0	p*	$\frac{\mathrm{T}1\hbox{-} \mathrm{T}0}{\mathrm{Max}}\left(\%\right)$
LL MEP score (/10)	3.6 (3.2)	5.5 (3.6)	1.9 (2.8)	<0.0001	18.8
Global EP score (/30)	11.5 (7.0)	14.2 (7.5)	2.7 (3.9)	<0.0001	8.9
LL SEP score (/10)	4.3 (3.3)	5.1 (3.6)	0.75 (2.0)	0.0019	7.5
UL MEP score (/10)	2.4 (2.8)	3.2 (3.3)	0.75 (2.8)	0.021	7.5
VEP score (/10)	4.2 (3.5)	4.9 (3.5)	0.61 (2.2)	0.017	6.1
EDSS (/10)	2.9 (1.1)	3.4 (1.6)	0.58 (1.1)	<0.0001	5.8
pFS (/6)	2.1 (1.1)	2.4 (1.2)	0.30 (0.91)	0.0049	5.0
vFS (/6)	0.80 (1.1)	1.1 (1.0)	0.26 (0.98)	0.023	4.4
sFS (/6)	1.8 (0.93)	1.9 (0.95)	0.013 (0.97)	0.91	0.22

*MS* multiple sclerosis, *EP* evoked potentials, *UL* upper limb, *LL* lower limb, *MEP* motor evoked potentials, *SEP* somatosensory evoked potentials, *VEP* visual evoked potentials, *EDSS* expanded disability status scale, *mFS* pyramidal functional system, *sFS* sensory functional system, *vFS* visual functional system, *Max* maximal theoretical value for each individual score (i.e. 30 for Global EP score, 10 for individual EP score and EDSS, 6 for functional system)

* *p*-value of the evolution between T0 and T1 (median follow-up = 6.3 years)

As seen in Table 4, the cross-sectional correlations (T0 and T1) between the different EP scores and EDSS or their related functional systems were all significant. The best association concerned the aggregate scores: global EP score and EDSS (*r*_s_ = 0.67, *p* < 0.001). Longitudinally (T0 → T1), the only significant correlation was attributed to the motor function for a follow-up period longer than 6.3 years (T0 → T1).

**Table 4:** Cross-sectional (T0, T1) and longitudinal (T0→T1) correlations (Spearman correlation) between electrophysiological and clinical data

	T0	T1		T0→T1	T0→T1 (≤6,3 y)	T0→T1 (>6,3 y)
	(*n*=100)	(*n*=76)		(*n*=76)	(*n*=38)	(*n*=38)
EDSS vs UL MEP	0.47 ***	0.49 ***	∆ EDSS vs ∆ UL MEP	0.20	0.08	0.31
EDSS vs LL MEP	0.62 ***	0.60 ***	∆ EDSS vs ∆ LL MEP	0.16	−0.13	0.44 **
EDSS vs LL SEP	0.61 ***	0.54 ***	∆ EDSS vs ∆ LL SEP	0.11	0.05	0.19
EDSS vs VEP	0.33 ***	0.34 **	∆ EDSS vs ∆ VEP	0.10	0.17	0.05
EDSS vs Global EP	0.67 ***	0.66 ***	∆ EDSS vs ∆ Global EP	0.18	0.07	0.28
pSF vs UL MEP	0.52 ***	0.56 ***	∆ pSF vs ∆ UL MEP	0.24 *	0.16	0.34 *
pSF vs LL MEP	0.56 ***	0.58 ***	∆ pSF vs ∆ LL MEP	0.16	−0.05	0.38 *
sSF vs LL SEP	0.53 ***	0.50 ***	∆ sSF vs ∆ LL SEP	0.18	0.12	0.25
vSF vs VEP	0.38 ***	0.40 ***	∆ vSF vs ∆ VEP	0.17	0.36 *	0.01

* *p* ≤ 0.05 ** *p* < 0.01 *** *p* < 0.001; 6.3 y = median follow-up in years in considered patients; *EP* evoked potentials, *UL* upper limb, *LL* lower limb, *MEP* motor evoked potentials, *SEP* somatosensory evoked potentials, *VEP* visual evoked potentials, *EDSS* expanded disability status scale, *mFS* pyramidal functional system, *sFS* somatosensory functional system; visual functional system; ∆, delta EDSS

**Fig. 1:**
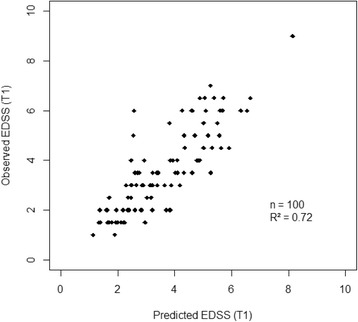
Correlation between observed and predicted EDSS at T1 from model 1. EDSS, Expanded Disability Status Scale

When predicting EDSS at T1 from baseline covariates by multiple regression analysis (model 1), only the global EP score and EDSS at baseline turned out to be significant after adjusting for follow-up time. None of the other parameters (e.g. demographic parameters, phenotype, disease duration, follow-up duration, disease-modifying therapy) were predictive of the outcome (Table 5). The strong correlation (*R*^2^ = 0.72) between observed and predicted EDSS from model 1 at T1 evidenced the high performance of the disability prediction (Fig. 1). When discarding EDSS from the model 1, the global EP score still explained more than half (*R*^2^ = 0.61) of the variance of EDSS at T1. Now, when considering the annual progression of EDSS (model 2), the global EP score at baseline was the only significant predictive parameter but the quality of the model was much lower (*R*^2^ = 0,21) (Table 5).

**Table 5:** Prediction models of EDSS (model 1) and annual EDSS progression (model 2) from baseline data

	Model 1		Model 2	
	EDSS		EDSS progression/year	
	(*n*=100, R^2^=0.72)		(*n*=100, R^2^=0.21)	
Baseline Predictor	Regression coefficient ± SE	*p*-value	Regression coefficient ± SE	*p*-value
Intercept	2.8 ± 3.3	0.39	0.33 ± 0.59	0.58
Age (yrs)	−0.0033 ± 0.014	0.81	−0.00001 ± 0.0025	0.99
Height (cm)	−0.015 ± 0.021	0.48	−0.0015 ± 0.0038	0.69
Weight (kg)	−0.0059 ± 0.0098	0.55	−0.0015 ± 0.0018	0.39
Sex (1= male)	0.090 ± 0.31	0.77	0.028 ± 0.056	0.62
Laterality (1=left)	−0.066 ± 0.38	0.86	−0.046 ± 0.070	0.52
Phenotype (1 = SP or PP)	0.39 ± 0.46	0.40	0.010 ± 0.083	0.90
Disease duration (yrs)	0.0054 ± 0.016	0.73	0.0027 ± 0.0029	0.35
Follow-up duration (yrs)	0.036 ± 0.046	0.43	NA	NA
DMT (1 = yes)	0.18 ± 0.37	0.63	0.057 ± 0.066	0.39
**EDSS**	**0.75 ± 0.13**	**<0.0001**	−0.035 ± 0.025	0.16
**EP Score**	**0.082 ± 0.018**	**<0.0001**	**0.012 ± 0.0034**	**0.0006**

*EDSS* expanded disability status scale; *R* ^2^ multiple determination coefficient, *SE* standard error, *SP* secondary progressive, *PP* primary progressive, *DMT* disease-modifying therapy during the follow-up, *EP* evoked potentials, *NA* not applicable. Baseline predictors with *p*-value < 0.05 are highlighted in bold

**Fig. 2:**
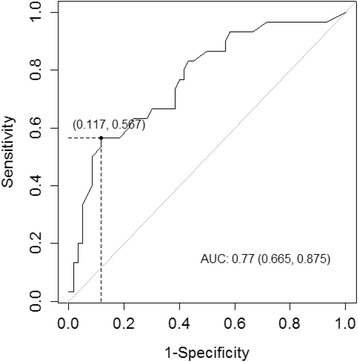
ROC curve of baseline global EP score with respect to EDSS worsening. Area under the ROC curve (AUC = 0.77) and best cut-off point (Global EP score = 17/30) with a sensitivity of 56.7 % and a specificity of 88.3 %. EDSS worsening (Yes/No) was defined as a 1-point increase from T0 to T1 given a baseline EDSS <5.5 or an increase of 0.5 point given a baseline EDSS ≥5.5

Among the 100 MS patients at baseline, 38 evidenced an EDSS worsening during follow-up and 89 % received a disease-modifying therapy between T0 and T1. Logistic regression analysis (model 3) was applied to evaluate the probability of disability worsening based on the same demographic, clinical and electrophysiological factors (Table 6). When accounting for follow-up duration, only the baseline global EP score was found to be of significant prognostic value (OR = 1.2; 95 % CI 1.1–1.3; *p* = 0.0012). Interestingly, the baseline EDSS (*p* = 0,24) did not impact the prediction, as for the factor therapy (binary variable) between T0 and T1 (*p* = 0,05). The prognostic efficacy of model 3 was high (AUC = 0.84). When predicting EDSS worsening by the sole global EP score at baseline, the AUC was 0.77, only slightly less than for the full model. Further, a global EP score of 17 (over a scale of 30) was determined as the best cut-off point at baseline to identify patients at high risk of EDSS aggravation (Fig. 2). This cut-off yielded a sensitivity of 56.7 %, a specificity of 88.3 % with a corresponding positive predictive value (PPV) of 70.8 %.

**Table 6:** Prediction model of EDSS worsening from baseline data

	Model 3 EDSS worsening (*n*=100, AUC=0.84)		
Baseline Predictor	Coefficient ± SE	*p*-value	OR (95 % CI)
Intercept	−0.87 ± 8.4	0.92	
Age (yrs)	0.0016 ± 0.035	0.97	
Height (cm)	−0.032 ± 0.053	0.55	
Weight (kg)	0.0057 ± 0.027	0.83	
Sex (1= male)	0.34 ± 0.79	0.67	
Laterality (1=left)	−0.34 ± 0.99	0.73	
Phenotype (1 = SP or PP)	0.78 ± 1.2	0.53	
Disease duration (yrs)	0.12 ± 0.048	0.01	
Follow-up duration (yrs)	0.029 ± 0.12	0.82	
DMT (1 = yes)	3.1 ± 1.6	0.05	
**EDSS**	−0.42 ± 0.36	0.24	0.66 (0.32–1.3)
**EP Score**	**0.18 ± 0.054**	**0.0012**	**1.2 (1.1–1.3)**

*EDSS* expanded disability status scale, *AUC* area under the ROC Curve, *OR* odds Ratio, *CI* confidence interval, *EP* evoked potentials, EDSS worsening, 1-point increase from T0 to T1 given a baseline EDSS <5.5 or an increase of 0.5 point given a baseline EDSS ≥ 5.5; DMT, disease-modifying therapy during the follow-up. Baseline predictors with *p*-value < 0.01 are highlighted in bold

## Discussion

For the past 30 years, MS diagnosis, disease activity monitoring, and clinical trials have been essentially based on clinical and conventional MRI data. At the individual follow-up level, though, there is an apparent paradox between clinical metrics and MRI T2 lesion load [12]. This paradox is due to a subclinical activity before the first clinical symptoms in the early stage of the disease [32]. Up to 50–70 % of patients with a clinically isolated syndrome (CIS) exhibit clinically silent T2-weighted lesions [33]. After the relapsing-remitting phase, disability progresses without any MRI focal inflammatory activity due to axonal loss and diffuse inflammatory infiltration, which is not visible through conventional MRI [34–36].

Electrophysiology provides functional quantified data on multimodal afferent and efferent pathways through the brain and long spinal tracks. A standardized procedure was developed to study highly relevant functions (VEP, LL SEP and 4 limbs MEP). We advocate that our EP battery (VEP, UL and LL MEP and LL SEP), which takes 90–120 min depending on the patient’s compliance, constitutes the best trade-off between time efficiency and information relevance. As mentioned previously, to improve the EP prognostic yield, it seems more important adding EP modalities than repeating electrophysiological evaluations within a short period of time [37]. In our study, a post hoc analysis designed to investigate the individual contribution of EP modalities showed that restricting the test battery to VEP and UL MEP did not significantly lowered the predictive power of our models. This observation is in line with Schlaeger et al. who stated that, for the long term disability prediction, VEP and UL MEP were the most relevant variables [38]. However, in another study aimed to prognosticate the future disability in a sample of primary progressive patients, the same authors claimed that multimodal EP (VEP, UL and LL MEP, UL and LL SEP) could be simplified to a test battery containing only UL MEP and LL SEP without loss of information [19]. From these observations, it appears that the superiority of one EP modality over another in the prognostic field depends on the population characteristic in terms of phenotype, disability and disease duration at the time of the electrophysiological assessment.

Latency, conduction times, and amplitude parameters were taken into account to evaluate demyelination and axonal loss or conduction blocks. We constructed a composite neurophysiological score and retrospectively applied it to 100 MS patients followed for a median duration of about 6 years. Cross-sectional correlations with EDSS were good, in agreement with those reported in previous studies [11, 16–22, 27, 39]. The global EP score in the progressive form (19.8/30) was significantly higher than in the RR form (11.2/30) regardless of disease duration, and was closely related to clinical metrics (Table 2). This difference is mainly attributed to motor and somatosensory EP, suggesting a higher implication of spinal cord dysfunction in the progressive phenotype [40]. The lower correlation between VEP and EDSS also confirms that EDSS overweighs ambulation and spinal cord metrics and relatively underweighs more advanced measures of visual disability like change in low contrast vision, colour vision or visual field. We confirmed the markedly weaker longitudinal correlations already noticed by other authors [11, 17, 20, 23]. The relative longitudinal progression rate of clinical data illustrates the low EDSS sensitivity to change with a nearly twice higher deterioration of the global EP score and LL MEP score compared to EDSS and the functional systems (Table 3). The weaker correlation in the subgroup with the shorter follow-up is probably an additional argument for the lower EDSS sensitivity. The EDSS scale needs a sufficient amount of time to measure functional changes detected at an earlier stage by electrophysiology. On the other hand, high EP score level or progression, with long track dysfunction, is not necessarily associated with a clinical deterioration due to plasticity or adaptation but certainly indicates a depletion of functional reserve [41].

This ability of EP to anticipate a clinical deterioration constitutes the most salient result of our study. In the past, models with EP alone [37, 39] or integrated in multivariate approaches [19, 20, 38, 42] were used to forecast disability and disability progression for relatively homogenous samples [19, 22, 37, 43]. In this context, we performed a multivariate analysis to define a model able to predict the EDSS score at 6 years. Predicted and observed EDSS were highly correlated in agreement with published data obtained in smaller groups and in specific conditions [19, 37]. The statistical analysis showed that the initial global EP score and EDSS explain a significant part of the variance of the EDSS, 6 years later. Furthermore, the baseline global EP score was the unique significant factor able to predict the EDSS annual progression rate (*p* < 0.001) and the risk of EDSS worsening after a median follow up period of 6.3 years (*p* < 0.005). Our study also provides an EP score cut-off limit of 17, on a scale ranging from 0 to 30, which is potentially useful at the individual level as it suggests that a patient with a Global EP score higher than 17/30 exhibits a risk of clinical worsening of around 70 %. To some extent, this observation provides a proof of the concept that electrophysiology could be used as part of a prognostic armamentarium in MS.

The present study was limited by the retrospective design and by the heterogeneity of the follow-up duration between subjects. Nevertheless, the multivariate analysis showed that this variability didn’t impact outcome prediction. While the therapeutic factor did not significantly impact the prognosticating potential of our EP score, as previously argued by Schlaeger on a smaller sample size [42], it would be valuable, in the era of highly active therapies, to design a prospective study focused on the prognostic interplay between EP and immunomodulatory drugs. Further studies are however needed to implement spinal and cerebral MRI metrics as well as to confirm the validity of our models, based on the same EP procedure but with other datasets. It could be useful, and not redundant, to integrate clinical, neurophysiological functional data and anatomical information to define a multivariate prognostic index.

Furthermore, EP amplitude measures can indirectly reflect central axonopathy, which is highly correlated with irreversible disability. Motor triple stimulation technique can precise and quantify corticospinal conduction failure and should be more largely implemented in the electrophysiological evaluation [44].

## Conclusion

In conclusion, multimodal EP, summarized in an ordinal score, seem to be well suited as a biomarker of the MS disease course given the high correlations observed with EDSS at any time point. Using a multivariate approach we proposed models for EDSS prediction and for the risk of clinical aggravation at a second follow-up time point. Based on ROC curve analysis we were able to define an EP score cut-off point which is associated with a high risk of disability progression. At the individual clinical level, this finding could be a relevant and independent argument for early highly active therapy. It could also support the process of patient selection in the scientific settings.

## Abbreviations

ANOVA, analysis of variance; AR, amplitude ratio; AUC, area under the ROC curve; CDMS, clinically definite diagnosis of MS; CI, confidence interval; CMAP, compound muscle action potential; CMCT, central motor conduction time; DMT, disease-modifying therapy; EDSS, expanded disability status scale; EP, evoked potentials; F, female; FDI, first dorsal interosseous; LL, lower limb; M, male; Max, maximal theoretical value for each individual score; MEP, motor evoked potentials; mFS, pyramidal functional system; MRI, magnetic resonance imaging; MS, multiple sclerosis; NA, not applicable; OR, odds ratio; P, progressive; PP, primary progressive; R^2^, coefficient of determination; ROC, receiver operating characteristic; RR, relapsing–remitting; r_s_, Spearman correlation coefficient; SD, standard deviation; SE, standard error; SEP, somatosensory evoked potentials; sFS, sensory functional system; SP, secondary progressive; TA, tibialis anterior; TMS, transcranial magnetic stimulation; UL, upper limb; VEP, visual evoked potentials; vFS, visual functional system.

## Additional file

Additional file 1: Raw demographical, clinical and electrophysiological data of the 100 MS patients obtained at T0 and T1. (XLSX 362 kb)
